# Battle Creek Sanitarium

**Published:** 1916-12

**Authors:** 


### Battle Creek Sanitarium.

To spend a few days at the Battle Creek Sanitarium is to get a new view of life; to spend a few months or weeks there is to get new life itself. At least that is the opinion of most people who go there.

The editor of the TEXAS MEDICAL JOURNAL spent a few days recently at the Sanitarium, and had ample opportunity to study the place closely. The marvelous success of the institution is due to a few things,—thorough diagnosis of all cases, dieting according to fixed standards, close watchfulness over patients, and getting the patients in the right mental attitude toward themselves and their disease. Added to these perhaps might be that word now so popular everywhere, "efficiency."

Of course, everyone who goes to Battle Creek does not get well; many put off going until too late; some would die before following any definite regime of living; and many have made up their mind that they will not be cured. Then, however much skill may be practiced, some cases cannot be cured.

But in the constant accounting with Health on the credit side and Disease on the debit side, the books always show the assets far, far ahead of the liabilities.

When people find what is the matter with them and then quit imposing on their stomachs and get in the right mental attitude, disease has a mighty hard time making any progress, and has to beat a retreat. That is what happens at Battle Creek, where very little medicine is used, but where the other things are given the closest attention.

One attractive feature of the Battle Creek Sanitarium is that it does not seem like a sanitarium at all, but has all the appearance of a big resort hotel, where everything possible is done for the comfort and entertainment of its guests. With music and lectures and physical culture exercises and all kinds of popular and healthful entertainment along with pleasant associations, the task of curing the diseased, to which Dr. Kellogg and his host of associates are giving their lives, is made much easier as well as much more pleasant for those under treatment.

Physicians everywhere recommend the Battle Creek Sanitarium and send their patients there, feeling assured that they get the best attention and treatment which can possibly be given.

---

Do you go to the drug store to buy a toothbrush and then handle the entire stock to see if the bristles are right?

